# Supplementary material for: Microglial cannabinoid receptor type 1 mediates social memory deficits in mice produced by adolescent THC exposure and 16p11.2 duplication
Source: Nat Commun. 2023 Oct 25;14:6559. doi: 10.1038/s41467-023-42276-5 (PMC10600150; doi:10.1038/s41467-023-42276-5)
Supplement: Supplementary file 1 — Supplementary Information [file 41467_2023_42276_MOESM1_ESM.pdf]

Supplementary Information

Microglial cannabinoid receptor type 1 mediates social memory deficits in mice produced by adolescent THC exposure and 16p11.2 duplication

Yuto Hasegawa[1], Juhyun Kim[1,2], Gianluca Ursini[1,3], Yan Jouroukhin[4], Xiaolei Zhu[1], Yu Miyahara[1], Feiyi Xiong[1], Samskruthi Madireddy[1], Mizuho Obayashi[1], Beat Lutz[5,6], Akira Sawa[1,7,8,9,10,11], Solange P. Brown[7,12], Mikhail V. Pletnikov[4,#], and Atsushi Kamiya[1,#]

[1]Department of Psychiatry and Behavioral Sciences, [7]Solomon H. Snyder Department of Neuroscience, [8]Biomedical Engineering, [9]Genetic Medicine, [10]Pharmacology and Molecular Sciences, Johns Hopkins University School of Medicine, Baltimore, MD, USA

[2] Korea Brain Research Institute, Daegu, Republic of Korea

[3]Lieber Institute for Brain Development, Johns Hopkins Medical Campus, Baltimore, MD, USA

[4]Department of Physiology and Biophysics, Jacobs School of Medicine and Biomedical Sciences SUNY, University at Buffalo, Buffalo, NY, USA

[5]Institute of Physiological Chemistry, University Medical Center of the Johannes Gutenberg University, Mainz, Germany

[6]Leibniz Institute for Resilience Research (LIR) gGmbH, Mainz, Germany

[11]Department of Mental Health, Johns Hopkins University Bloomberg School of Public Health, Baltimore, MD, USA

[12]Kavli Neuroscience Discovery Institute, Johns Hopkins University, Baltimore, MD, USA

[#]Correspondence: akamiya1@jhmi.edu (A.K.) and mvpletni@buffalo.edu (M.V.P.)

Supplementary Fig. 1

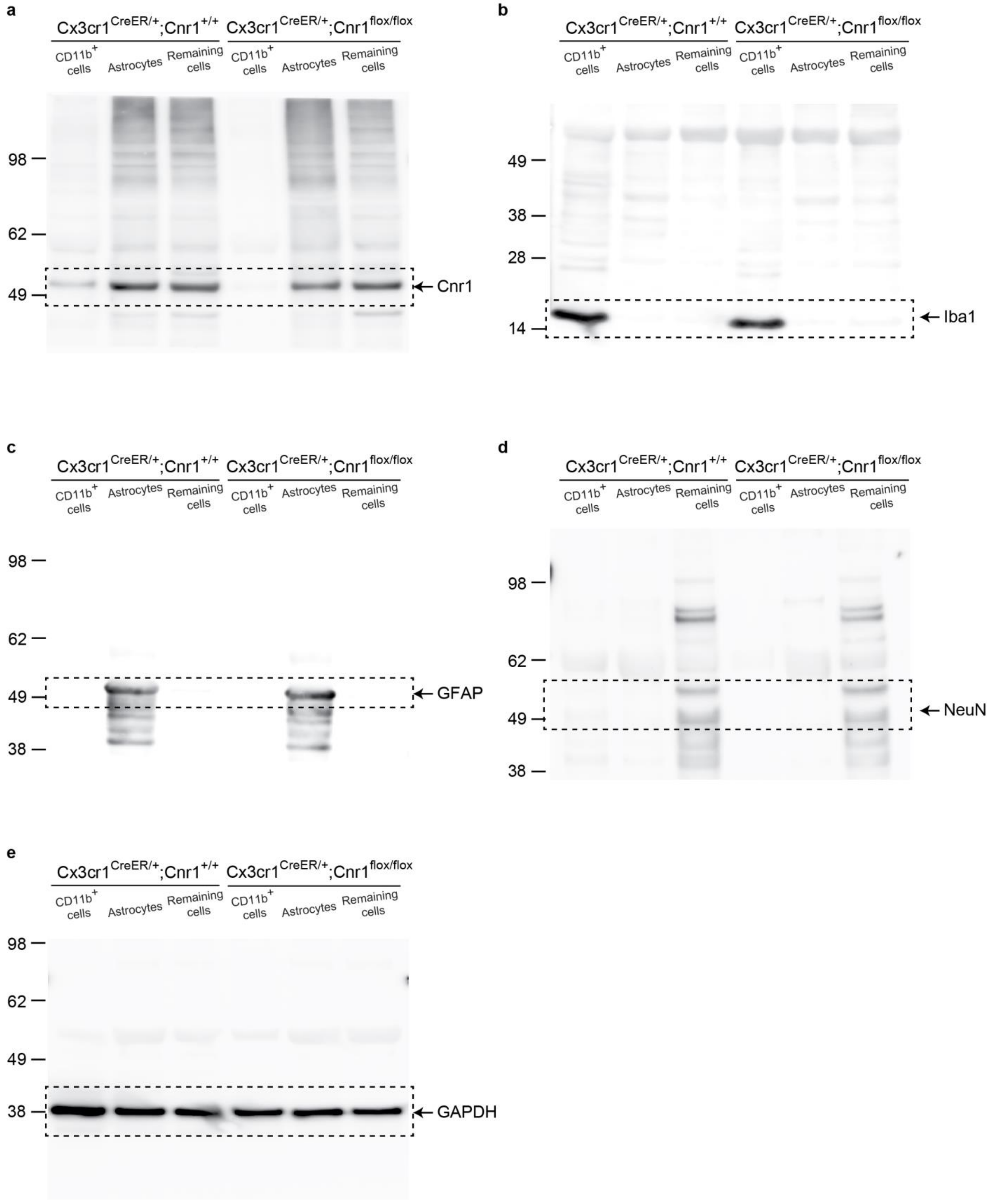

Supplementary Fig. 1 The full scan images corresponding to Fig. 1d. Microglia-enriched $CD11b^+$ cells, ACSA-2^+ astrocytes, and remaining cells were collected from the cerebral cortex of *Cx3cr1*$^{CreER/+}$;*Cnr1*$^{flox/flox}$ mice and littermate controls (*Cx3cr1*$^{CreER/+}$;*Cnr1*$^{+/+}$) using magnetic activated cell sorting (MACS). The protein expression of Cnr1(a), Iba1 (b), GFAP (c), NeuN (d), and GAPDH (e) that correspond to Fig. 1d is shown within the rectangles outlined by dotted lines of the full-scan images.

Supplementary Fig. 2

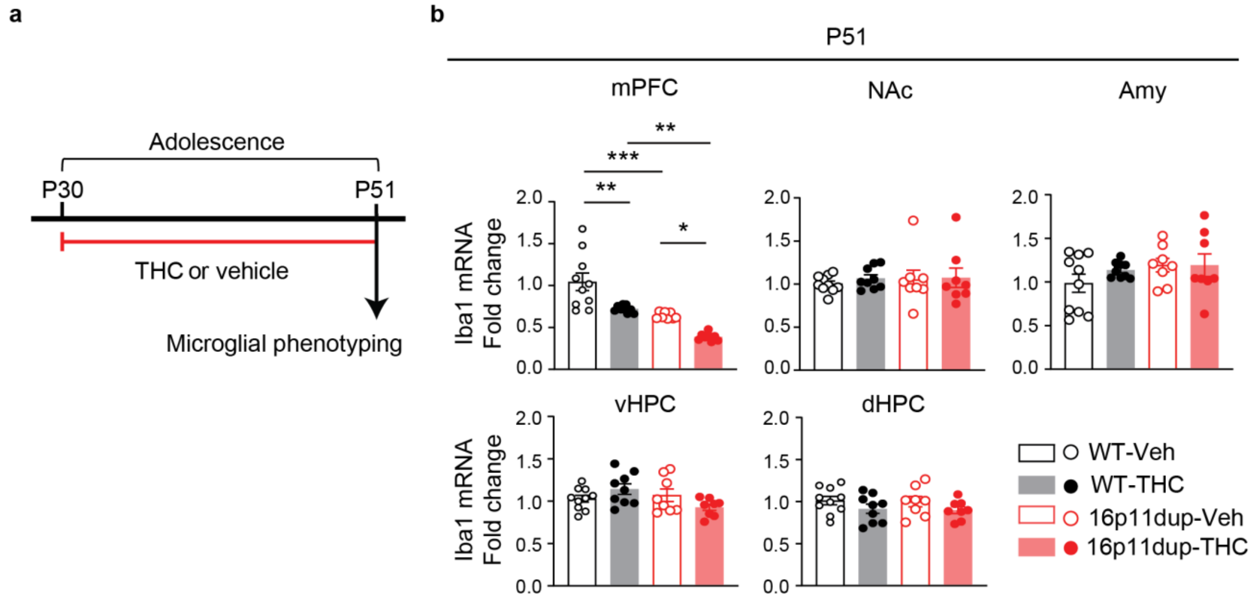

Supplementary Fig. 2 Iba1 mRNA reduction in the mPFC produced by adolescent THC treatment and 16p11dup. a, Schematic diagram of the adolescent THC treatment protocol. 16p11dup mice and wild type littermate controls (WT) were treated with THC (s.c., 8mg/kg) or vehicle (Veh) during adolescence (P30-P51), followed by microglial phenotyping at P51 upon completion of THC treatment. **b,** Relative mRNA expression level of Iba1 in brain regions involved in the medial prefrontal cortex (mPFC), ventral hippocampus (vHPC), dorsal hippocampus (dHPC), nucleus accumbens (NAc), and amygdala (Amy) at P51. WT-Veh (n = 10 mice), WT-THC (n = 9 mice), 16p11dup-Veh (n = 8 mice), and 16p11dup-THC (n = 8 mice). (**b**) ***$p < 0.001$, **$p < 0.01$, *$p < 0.05$ (p values are WT-Veh versus WT-THC: $p = 0.0012$, WT-Veh versus 16p11dup-Veh: $p = 0.0003$, WT-THC versus 16p11dup-THC: $p = 0.0047$, 16p11dup-Veh versus 16p11dup-THC: $p = 0.0447$), determined by two-way ANOVA with post hoc Tukey test. Each symbol represents one animal. Data are presented as the mean ± s.e.m.

Supplementary Fig. 3

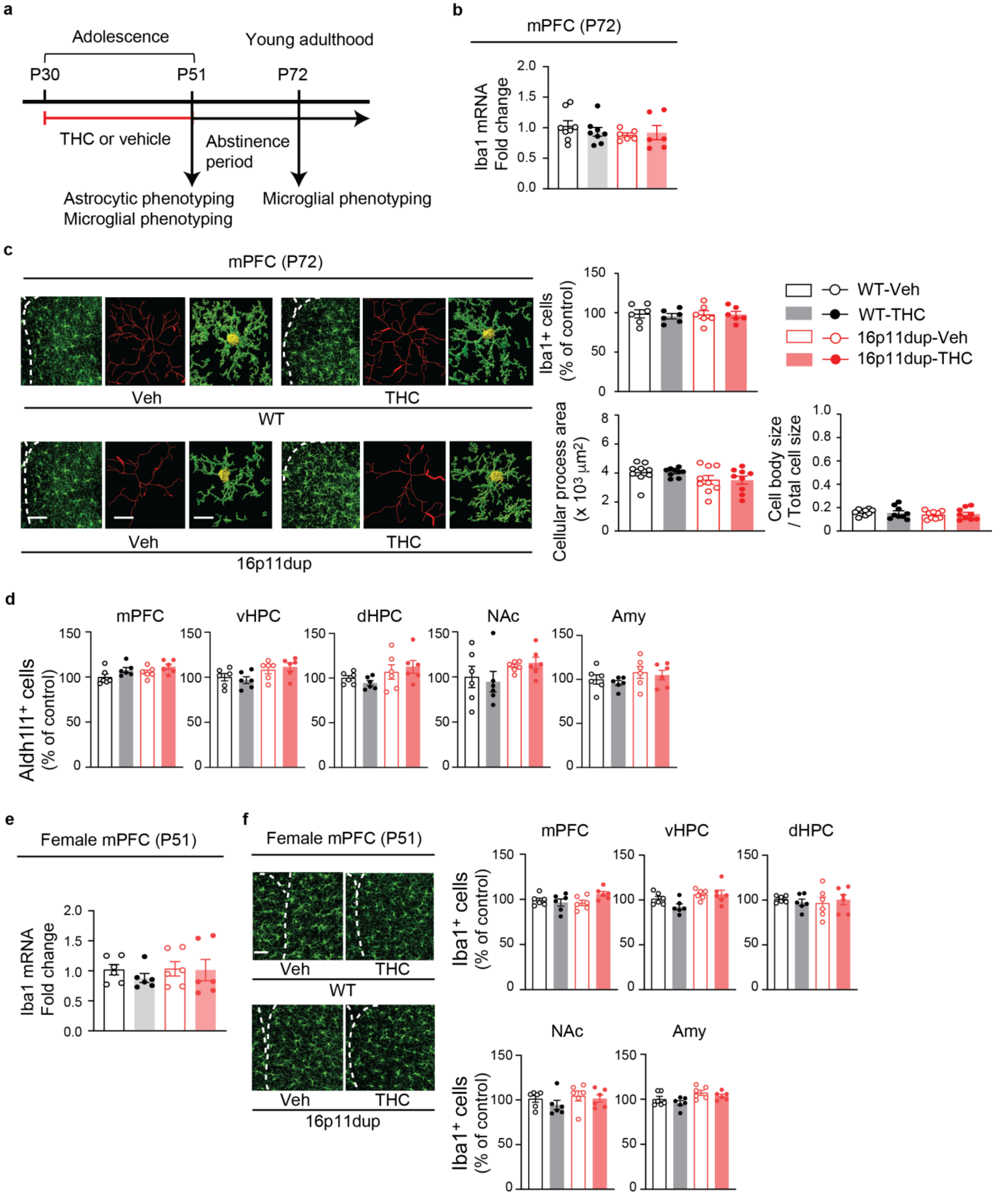

Supplementary Fig. 3 No effect of adolescent THC treatment and 16p11dup on microglial phenotypes in adulthood, the number of astrocytes, and female microglial phenotypes. a, Schematic diagram of the adolescent THC treatment protocol. 16p11dup mice and wild type littermate controls (WT) were treated with THC (s.c., 8mg/kg) or vehicle (Veh) during adolescence (P30-P51), followed by astrocytic phenotyping and microglial phenotyping at P51 upon completion of THC treatment and microglial phenotype at P72 after a 3-week abstinence period. **b**, Relative mRNA expression level of Iba1 in the medial prefrontal cortex (mPFC) at P72. WT-Veh (*n* = 8 mice), WT-THC (*n* = 8 mice), 16p11dup-Veh (*n* = 6 mice), 16p11dup-THC (*n* = 6 mice). **c,** Immunohistochemistry of Iba1 (green) in the mPFC at P72. (Left) Representative images of the mPFC, representative tracing images (red) along with images of cellular processes (green) and cell bodies (yellow) of $Iba1^+$ cells. Scale bar, 50 μm (left) and 10 μm (middle and right). (Top right) The number of $Iba1^+$ cells in the mPFC, presented as % of control. (*n* = 6 slices in 3 mice per condition). (Bottom right) Quantification of cellular process area (left) and the ratio of cell body size to total cell size (right) of $Iba1^+$ cells. (n = 9 cells in 3 mice per condition). **d,** Immunohistochemistry of Aldh1l1 in the mPFC, ventral hippocampus (vHPC), dorsal hippocampus (dHPC), nucleus accumbens (NAc), and amygdala (Amy) at P51. The number of $Aldh1l1^+$ cells in these brain regions, presented as % of control. (*n* = 6 slices in 3 mice per condition). **e**, Relative mRNA expression level of Iba1 in the mPFC of female mice at P51. (*n* = 6 mice per condition). **f,** Immunohistochemistry of Iba1 (green) in the mPFC, vHPC, dHPC, NAc, and Amy at P51. (Left) Representative images of the mPFC. Scale bar, 50 μm. (Right) The number of $Iba1^+$ cells in these brain regions, presented as % of control (*n* = 6 slices in 3 mice per group). Each symbol represents one animal (**b, e**), one slice (**c**-top, **d, f**), and one cell (**c**-bottom). Data are presented as the mean ± s.e.m.

Supplementary Fig. 4

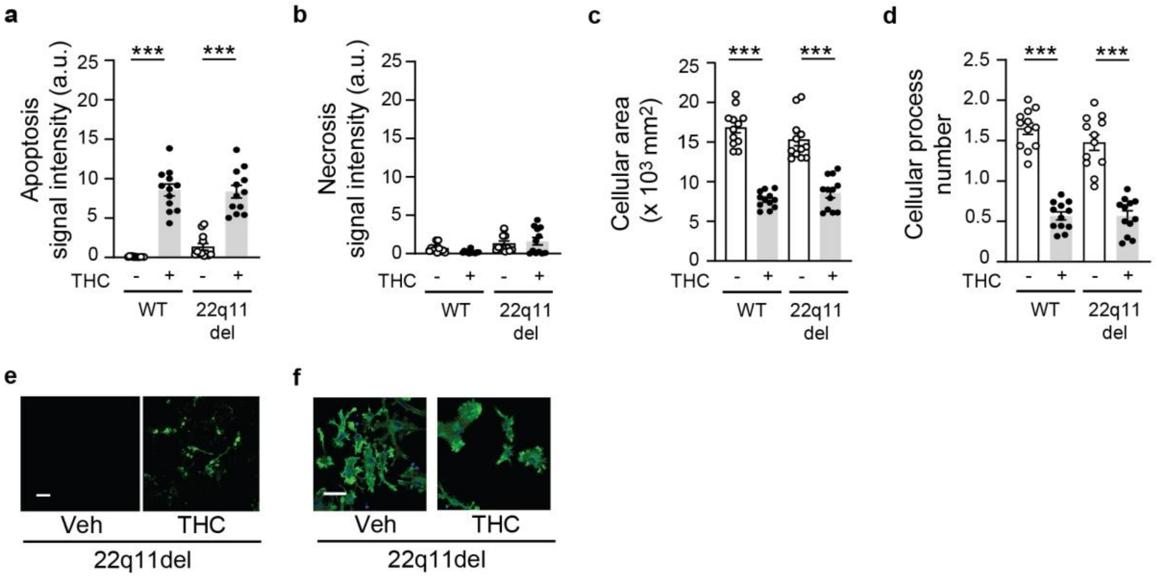

Supplementary Fig. 4 22q11del did not enhance THC-induced microglial phenotypes and p53 inhibition blocked microglial apoptosis produced by THC treatment and 16p11dup. a, Apoptosis assay of primary microglia cultures produced from WT and 22q11del male mice. Quantification of signal intensity (arbitrary units: a.u.) of apoptosis marker apopxin (n = 12 fields in 3 mice per condition). **b,** Necrosis assay of primary microglia cultures produced from WT and 22q11del male mice. Quantification of signal intensity (arbitrary units: a.u.) of necrosis marker 7-AAD (n = 12 fields in 3 mice per condition). **c,** Quantification of cellular area of phalloidin-stained microglia cultures produced from WT and 22q11del male mice (n = 12 fields in 3 mice per condition). **d**, Quantification of cellular process number of phalloidin-stained microglia cultures produced from WT and 22q11del male mice (n = 12 fields in 3 mice per condition). **e,** Representative images of microglia cell cultures produced from WT and 22q11del male mice in apoptosis and necrosis assays. Apopxin (green) and 7-AAD (red) are shown. Scale bar, 50 µm. **f,** Immunohistochemistry with antibody against phalloidin (green) of primary microglia cultures produced from WT and 22q11del male mice. Scale bar, 25 µm. (**a**, **c, d**) ***p < 0.001, **p < 0.01, *p < 0.05 (p values are all: p < 0.0001), determined by two-way ANOVA with post hoc Tukey test. Each symbol represents one field. Data are presented as the mean ± s.e.m.

Supplementary Fig. 5

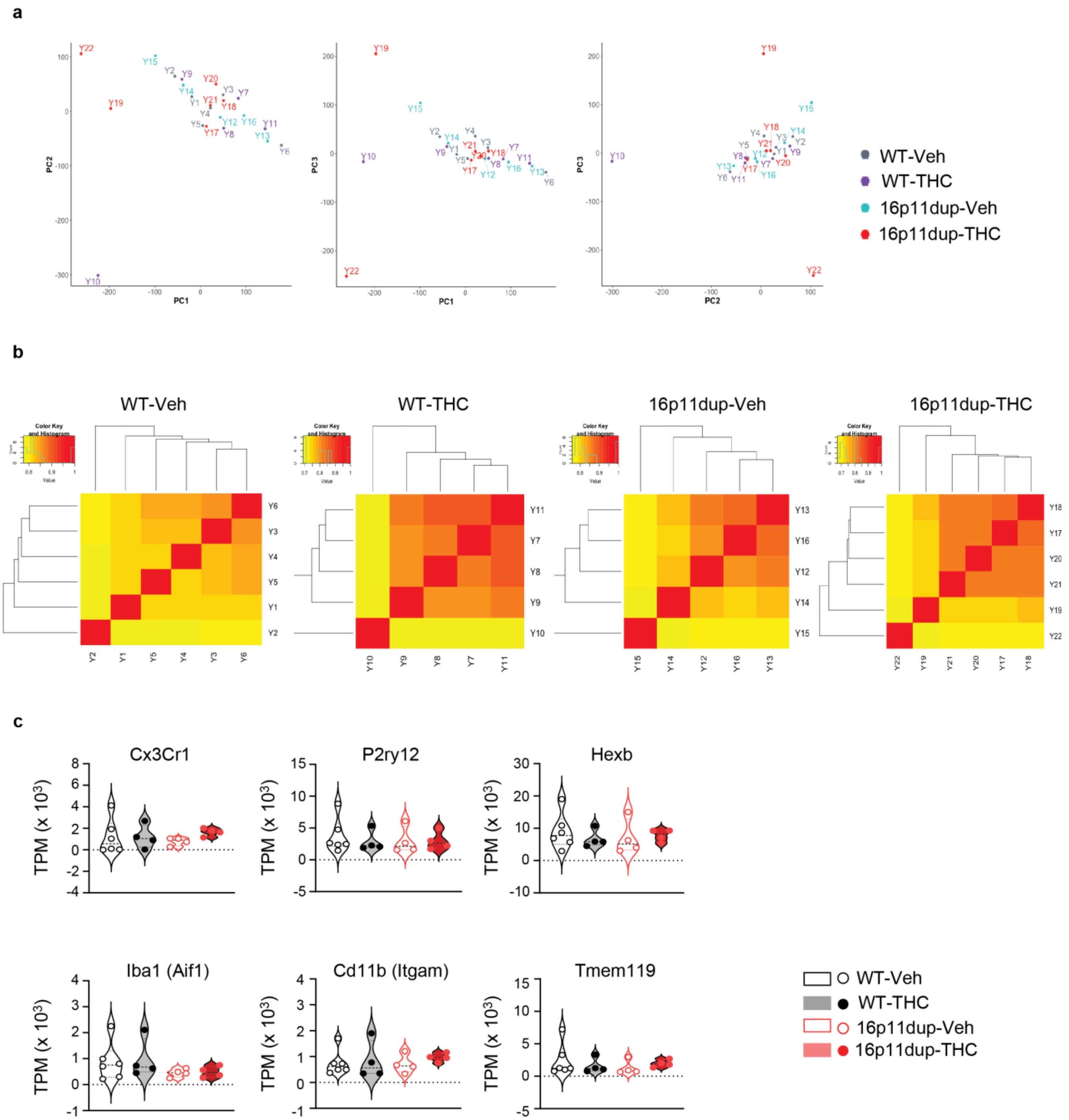

Supplementary Fig. 5 Quality control analysis for microglia-specific transcriptome profiling. a, Principal component analysis (PCA) plot showing, in all samples, the relationship between PC1 and PC2 (Left), PC1 and PC3 (Middle), PC2 and PC3 (Right). **b**, Heat maps of Pearson correlation coefficients for WT and 16p11dup mice with adult THC or Veh treatment. Samples detected as outliers (Y10, Y15, Y19, Y22) with Inter Array Correlation (see Methods) show lower correlation with the other biological replicates (**b**) and are clearly detectable in the PC plots (**a**). **c**, Violin plots of normalized gene expression level by transcripts per million (TPM) from RNA-seq data for 6 different microglia marker genes. (**c**) Each symbol represents one animal. Data are presented as the mean ± s.e.m.

Supplementary Fig. 6

a

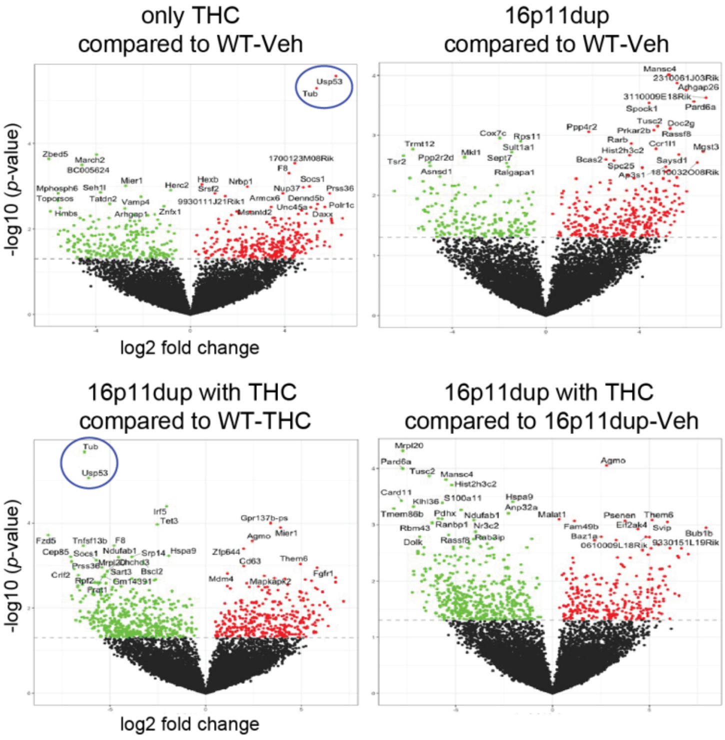

b

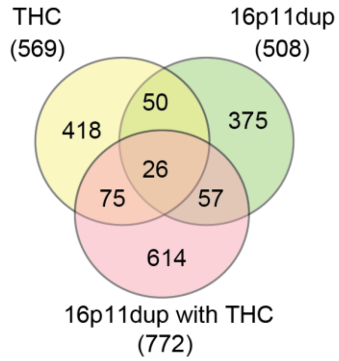

c

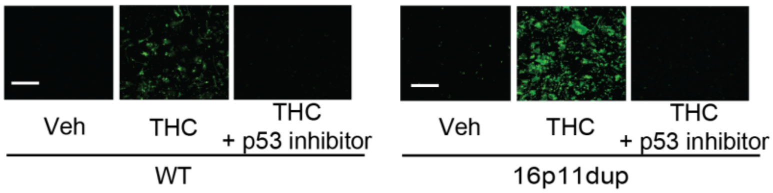

Supplementary Fig. 6 mPFC microglia-specific transcriptome profiling and p53 inhibition blocked microglial apoptosis produced by THC treatment and 16p11dup. a, Volcano plot representations of expression trends associated with THC alone, 16p11dup alone, and 16p11dup with THC compared to control, THC alone, and 16p11dup alone, showing down-regulated genes (green), up-regulated genes (red), and not associated genes (black), with uncorrected $p<0.05$. The differentially expressed genes (DEGs) with FDR<0.10 are highlighted using blue circles. **b**, Venn diagram showing the overlap of genes (with uncorrected $p<0.05$) associated with THC treatment or 16p11dup alone, and 16p11dup condition exposed to THC (16p11dup with THC), compared to controls (see also Supplementary Data 2 for full results from all the analyses). **c**, Representative images of primary microglia cultures treated by THC or vehicle together with and without pifithrin-α, an inhibitor of p53 transcriptional activity, in apoptosis and necrosis assays. Apopxin (green) and 7-AAD (red) were shown. Scale bar, 50 μm. (**a**, **b**) n = 4-6 mice per condition.

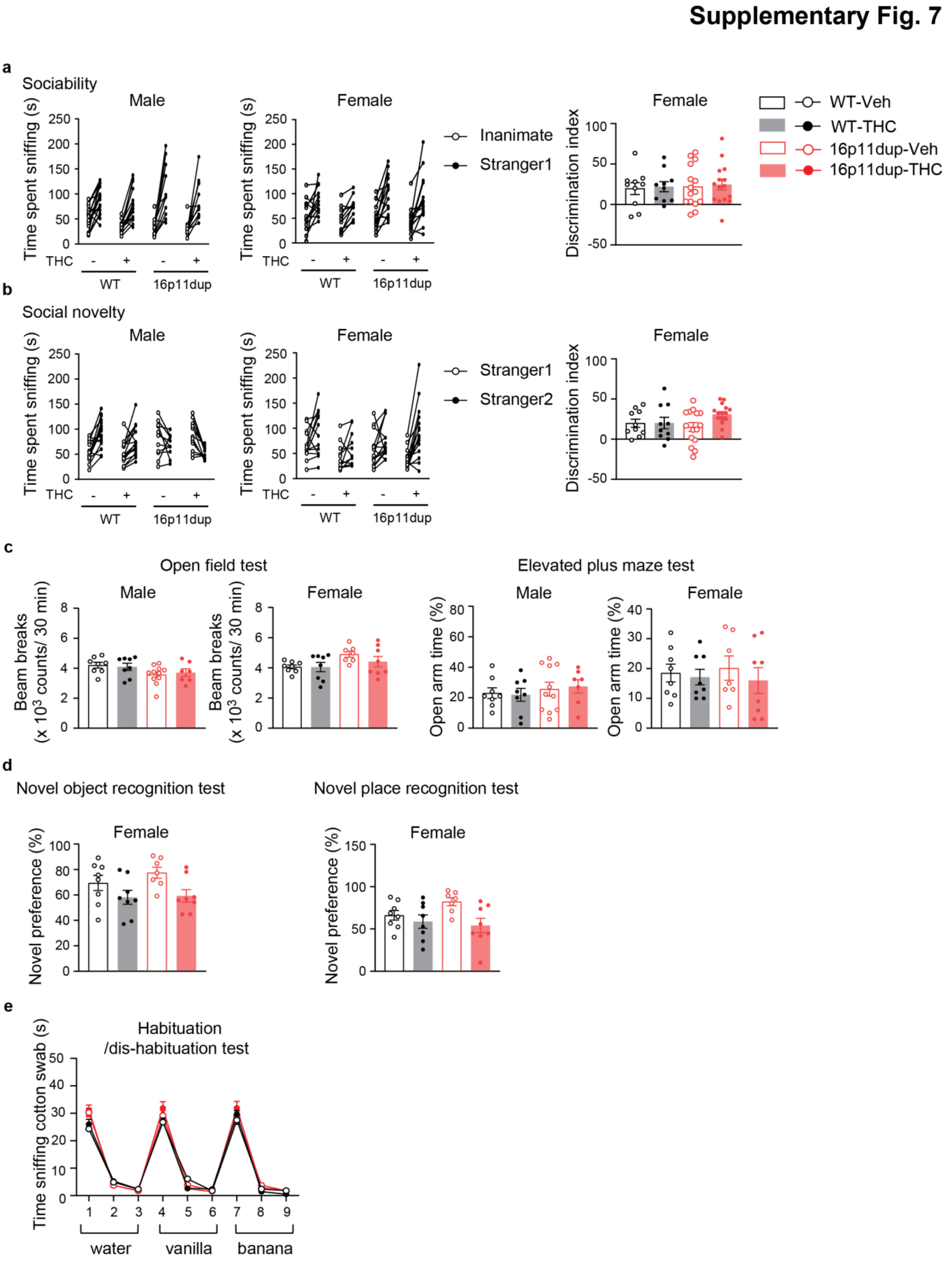
Supplementary Fig. 7
a
Sociability
Male
Female
Time spent sniffing (s)
THC
WT
16p11dup
Inanimate
Stranger1
Discrimination index
WT-Veh
WT-THC
16p11dup-Veh
16p11dup-THC
b
Social novelty
Stranger1
Stranger2
c
Open field test
Elevated plus maze test
Beam breaks
(x 10³ counts/ 30 min)
Open arm time (%)
d
Novel object recognition test
Novel place recognition test
Novel preference (%)
e
Habituation
/dis-habituation test
Time sniffing cotton swab (s)
water
vanilla
banana

Supplementary Fig. 7 No effect of adolescent THC treatment and 16p11 on spontaneous locomotion, anxiety, olfaction, or object and place recognition memory. a, (Left) Time the mice spent sniffing an Inanimate vs. a Stranger 1 mouse in male and female mice. (Right) Sociability of WT and 16p11dup female mice with adolescent THC or Veh treatment as indicated by discrimination index ([Stranger 1 – Inanimate mice sniffing time] / [Stranger 1 + Inanimate mice sniffing time] × 100 (%)). **b**, (Left) Time the mice spent sniffing a Stranger 1 mouse vs. a Stranger 2 mouse in male and female mice. (Right) Preference of social novelty of WT and 16p11dup female mice with adolescent THC or Veh treatment as indicated by discrimination index ([Stranger 2 – Stranger 1 mice sniffing time] / [Stranger 2 + Stranger 1 mice sniffing time] × 100 (%)). (**a**, **b**) Male: WT-Veh (n = 17 mice), WT-THC (n = 15 mice), 16p11dup-Veh (n = 11 mice), 16p11dup-THC (n = 11 mice); Female: WT-Veh (n = 10 mice), WT-THC (n = 10 mice), 16p11dup-Veh (n = 15 mice), 16p11dup-THC (n = 15 mice). **c**, (Left) Spontaneous locomotion activity of 16p11dup and wild type littermate (WT) male and female mice with adolescent THC or Veh treatment as indicated by total beam breaks in the open field test. (Right) Anxiety-like phenotype of WT and 16p11dup male and female mice with adolescent THC or Veh treatment as indicated by percentage of time spent in the open arms in the elevated plus maze test. **d**, (Left) Object recognition memory of WT and 16p11dup female mice with adolescent THC or Veh treatment as indicated by preference of novel object in the novel object recognition test. (Right) Place recognition memory of WT and 16p11dup female mice with adolescent THC or Veh treatment as indicated by preference of novel place in the novel place recognition test. (**c**, **d**) Male: WT-Veh (n = 8 mice), WT-THC (n = 8 mice), 16p11dup-Veh (n = 11 mice), 16p11dup-THC (n = 7 mice), Female: WT-Veh (n = 8 mice), WT-THC (n = 8 mice), 16p11dup-Veh (n = 7 mice), 16p11dup-THC (n = 8 mice). **e**, Olfactory discrimination in WT and 16p11dup mice with adolescent THC or Veh treatment as indicated by time the mice spent sniffing cotton swab in habituation/dis-habituation test. WT-Veh (n = 17 mice),

WT-THC (n = 15 mice), 16p11dup-Veh (n = 11 mice), 16p11dup-THC (n = 11 mice). Each symbol represents one field. Data are presented as the mean ± s.e.m.

Supplementary Fig. 8

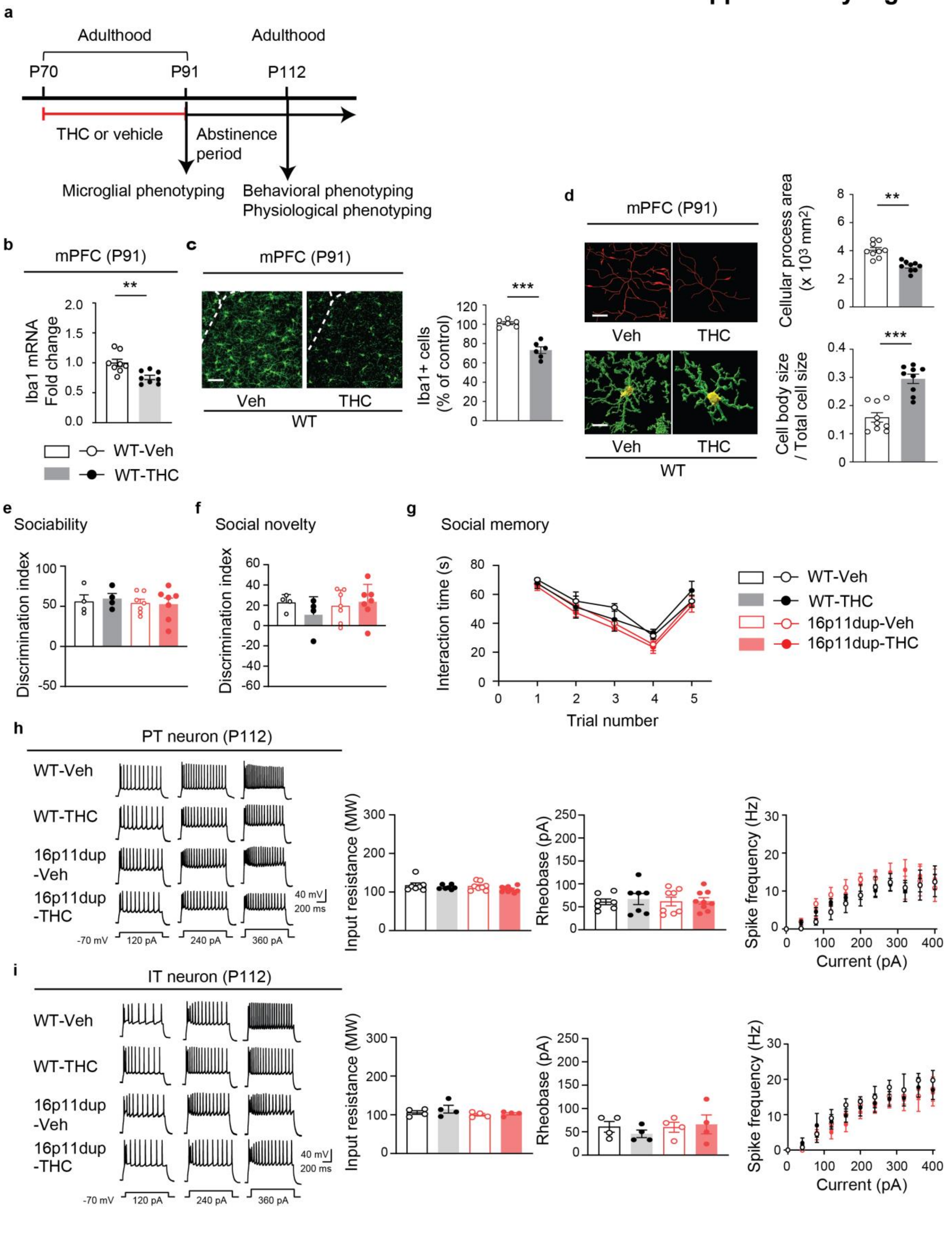

Supplementary Fig. 8 Adult THC treatment induced microglia reduction and morphological changes, but no effect on social recognition, memory and intrinsic excitability in mPFC. a, Schematic diagram of the adult THC treatment protocol. Wild type male mice (WT) were treated with THC (s.c., 8mg/kg) or vehicle (Veh) during adulthood (P70-P91), followed by microglial phenotyping at P91 upon completion of THC treatment and behavioral and physiological assays after a 3-week abstinence period. **b**, Relative mRNA expression level of Iba1 in the mPFC at P91. (*n* = 8 mice per condition). **c**, Immunohistochemistry of Iba1 (green) in the mPFC at P91. (Left) Representative images of the mPFC. Scale bar, 50 μm. (Right) The number of $Iba1^+$ cells in the mPFC, presented as % of control (*n* = 6 slices in 3 mice per condition). **d**, Microglial morphology analysis of individual $Iba1^+$ cells in the mPFC. (Top left) Representative tracing images (red) of $Iba1^+$ cells. Scale bar, 10 μm. (Top right) Quantification of cellular process area of $Iba1^+$ cells. (*n* = 9 cells in 3 mice per condition). (Bottom left) Representative images of cellular processes (green) and cell bodies (yellow) of $Iba1^+$ cells. Scale bar, 10 μm. (Bottom right) Quantification of the ratio of cell body size to total cell size of $Iba1^+$ cells (n = 9 cells in 3 mice per condition). **e,** Sociability of WT and 16p11dup mice with adult THC or Veh treatment as indicated by discrimination index ([Stranger1 – Inanimate mice sniffing time] / [Stranger 1 + Inanimate mice sniffing time] × 100 (%)). **f**, Preference of social novelty of WT and 16p11dup mice with adult THC or Veh treatment as indicated by discrimination index ([Stranger 2 – Stranger 1 mice sniffing time] / [Stranger 2 + Stranger 1 mice sniffing time] × 100 (%)). **g**, Interaction time of WT and 16p11dup mice receiving adult THC or Veh treatment with ovariectomized female mice. (**e, f, g**) WT-Veh (*n* = 4 mice), WT-THC (*n* = 4 mice), 16p11dup-Veh (*n* = 7 mice), 16p11dup-THC (*n* = 7 mice). **h,** (Left) Representative voltage traces recorded from PT neurons in response to current step injections in the presence of blockers of AMPA, NMDA, and GABAa receptors. (Right) The intrinsic excitability assessed by measurement of input resistance (left), rheobase (middle), and spike frequency (right). WT-

Veh (*n* = 7 cells in 4 mice), WT-THC (*n* = 7 cells in 3 mice), 16p11dup-Veh (*n* = 8 cells in 3 mice), and 16p11dup-THC (*n* = 9 cells in 3 mice). **i,** (Left) Representative voltage traces recorded from IT neurons in response to current step injections in the presence of blockers of AMPA, NMDA, and GABAa receptors. (Right) The intrinsic excitability assessed by measurement of input resistance (left), rheobase (middle), and spike frequency (right). WT-Veh (*n* = 4 cells in 2 mice), WT-THC (*n* = 4 cells in 2 mice), 16p11dup-Veh (*n* = 4 cells in 2 mice), and 16p11dup-THC (*n* = 4 cells in 3 mice). (**b, c, d**) ***$p < 0.001$, **$p < 0.01$ (*p* values are **b**, $p = 0.0042$; **c**, $p < 0.0001$; **d**, $P = 0.0039$, $P = 0.0004$), determined by unpaired two-tailed Student *t*-tests. Each symbol represents one animal (**b, e, f**), one slice (**c**), and one cell (**d, h, i**). Data are presented as the mean ± s.e.m.

Supplementary Fig. 9

a

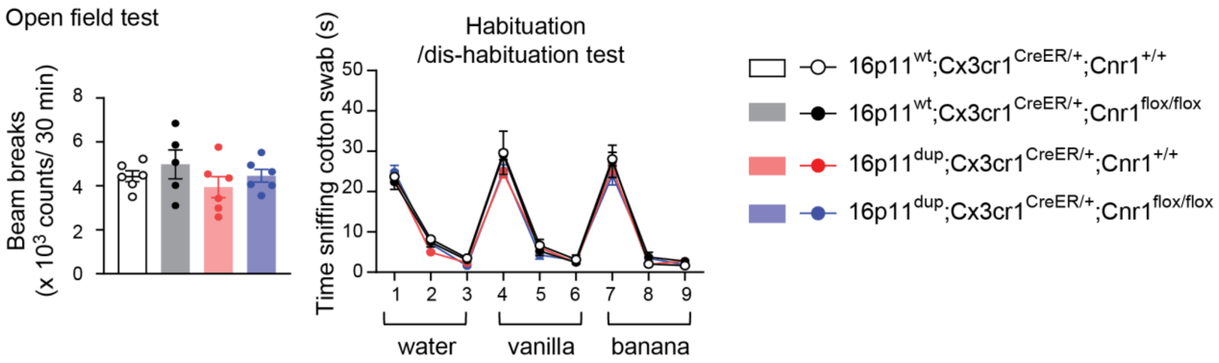

Supplementary Fig. 9 No effects of microglial genetic deletion of *Cnr1* on locomotion and olfaction in the male mice treated with THC during adolescence. a, (Left) Spontaneous locomotion activity of four groups of mice which include 16p11^{dup}; *Cnr1*$^{+/+}$, 16p11^{dup};*Cnr1*$^{flox/flox}$, wild type littermate (16p11^{wt});*Cnr1*$^{+/+}$, and 16p11^{wt} ;*Cnr1*$^{flox/flox}$ mice with adolescent THC treatment as indicated by total beam breaks in the open field test. (Right) Olfactory discrimination of these four groups of mice with adolescent THC treatment as indicated by time that the mice spent sniffing cotton swab in habituation/dis-habituation test. 16p11^{dup};*Cnr1*$^{+/+}$ (*n* = 6 mice), and 16p11^{dup};*Cnr1*$^{flox/flox}$ (*n* = 5 mice), 16p11^{wt};*Cnr1*$^{+/+}$ (*n* = 6 mice), and 16p11^{wt} ;*Cnr1*$^{flox/flox}$ (*n* = 6 mice). Each symbol represents one animal (Left), and averaged data per group (Right). Data are presented as the mean ± s.e.m.

Supplementary Data list

Supplementary Data 1: Sample information and gene expression data (CPM)

Supplementary Data 2: Full statistics from the differential gene expression analyses for each comparison

Supplementary Data 3: Enriched pathway and upstream regulator list
