# Supplementary material for: Microglial cannabinoid receptor type 1 mediates social memory deficits in mice produced by adolescent THC exposure and 16p11.2 duplication
Source: Nat Commun. 2023 Oct 25;14:6559. doi: 10.1038/s41467-023-42276-5 (PMC10600150; doi:10.1038/s41467-023-42276-5)
Supplement: Supplementary file 2 — Description of Additional Supplementary Files [file 41467_2023_42276_MOESM2_ESM.pdf]

Description of Additional Supplementary Files

File Name: Supplementary Data 1
Description: Sample information and gene expression data (CPM)

File Name: Supplementary Data 2
Description: Full statistics from the differential gene expression analyses for each comparison

File Name: Supplementary Data 3
Description: Enriched pathway and upstream regulator list
